# The Humoral Immune Response of the ChAdOx1 nCoV-19 Vaccine in Maintenance Dialysis Patients without Prior COVID-19 Infection

**DOI:** 10.3390/vaccines10020338

**Published:** 2022-02-21

**Authors:** Chung-Yi Cheng, Te-Chao Fang, Hung-Wei Liao, Tso-Hsiao Chen, Jer-Hwa Chang, Yen-Chung Lin, Chih-Chin Kao, Ming-Che Liu, Hui-Wen Chang, Ching-Sheng Hung, Jude Chu-Chun Wang, Shih-Hsin Hsiao, Yuh-Mou Sue

**Affiliations:** 1 Division of Nephrology, Department of Internal Medicine, School of Medicine, College of Medicine, Taipei Medical University, Taipei 11031, Taiwan; 94426@w.tmu.edu.tw (C.-Y.C.); fangtc@tmu.edu.tw (T.-C.F.); 88128@e.tmu.edu.tw (T.-H.C.); yclin0229@tmu.edu.tw (Y.-C.L.); salmonkao@gmail.com (C.-C.K.); 2 Division of Nephrology, Department of Internal Medicine, Wan Fang Hospital, Taipei Medical University, Taipei 11696, Taiwan; 3 Taipei Medical University-Research Center of Urology and Kidney (RCUK), School of Medicine, College of Medicine, Taipei Medical University, Taipei 11031, Taiwan; 4 Division of Nephrology, Department of Internal Medicine, Taipei Medical University Hospital, Taipei 11031, Taiwan; 5 Chinru Clinic, Taipei 116, Taiwan; lhw898@gmail.com; 6 School of Respiratory Therapy, College of Medicine, Taipei Medical University, Taipei 11031, Taiwan; m102094030@tmu.edu.tw; 7 Division of Pulmonary Medicine, Department of Internal Medicine, Wan Fang Hospital, Taipei Medical University, Taipei 11696, Taiwan; 8 Pulmonary Research Center, Wan Fang Hospital, Taipei Medical University, Taipei 11696, Taiwan; 9 School of Dental Technology, College of Oral Medicine, Taipei Medical University, Taipei 11031, Taiwan; d204097002@tmu.edu.tw; 10 Clinical Research Center, Taipei Medical University Hospital, Taipei 11031, Taiwan; 11 School of Medical Laboratory Science and Biotechnology, College of Medical Science and Technology, Taipei Medical University, Taipei 11031, Taiwan; g160090005@tmu.edu.tw (H.-W.C.); oryx@w.tmu.edu.tw (C.-S.H.); 12 Department of Laboratory Medicine, Taipei Medical University Hospital, Taipei 11031, Taiwan; 13 Department of Laboratory Medicine, Wan Fang Hospital, Taipei Medical University, Taipei 11696, Taiwan; 14 Division of Pulmonary Medicine, Department of Internal Medicine, School of Medicine, College of Medicine, Taipei Medical University, Taipei 11031, Taiwan; judewang1218@gmail.com; 15 Division of Pulmonary Medicine, Department of Internal Medicine, Taipei Medical University Hospital, Taipei 11031, Taiwan

**Keywords:** anti-receptor-binding-domain antibody, ChAdOx1 nCoV-19 (Oxford-AstraZeneca, AZ) vaccine, chronic dialysis, COVID-19 vaccine, immune response, SARS-CoV2

## Abstract

Background: Chronic kidney disease (CKD) patients tend to have a reduced immune response to infection and vaccination. The efficacy of current available COVID-19 vaccines in CKD patients has not been widely evaluated. Methods: In the present study, three hundred and eight chronic dialysis patients received ChAdOx1 nCoV-19 (Oxford-AstraZeneca, AZ). Blood tests using an antibody against the receptor-binding domain (RBD) of the S1 subunit of the SARS-CoV-2 spike protein had performed at four designed time points before and after the first and second vaccine. Results: The mean age of patients was 65.5 ± 12.38 years, and the male/female ratio was 61.4%:38.6% (189/119). Two weeks after the first vaccination, only 37.66% of patients had a positive antibody response (>50 AU/mL). However, 65.58% of the participants showed a delayed antibody response ten weeks after the first vaccine. Four weeks after the second vaccine, 94.16% of participants had positive antibody levels. Age was the most significant factor associated with antibody response. Flow cytometry analysis revealed that immune-naïve patients had significantly lower early active B cells and proliferative B cells than the age- and sex-matched immune responders. Conclusion: Despite a delayed response, 94.16% of chronic dialysis patients achieved a positive antibody response after two doses of the AZ vaccine. Age is the most significant factor associated with antibody response.

## 1. Introduction

Since the first case reported on 31 December 2019, the coronavirus disease 2019 (COVID-19) has continued to spread worldwide. More than 220 million infected cases have been reported and have caused more than five million deaths [1]. Chronic kidney disease (CKD) patients are vulnerable to COVID-19 and show higher morbidity and mortality than the general population [2,3,4,5,6]. Thus, prioritizing CKD patients for severe acute respiratory syndrome-coronaviruses-2 (SARS-CoV-2) vaccination has been at the forefront of vaccination programs internationally [7]. CKD patients tend to have a reduced immune response to vaccination [8]. Consequently, a need for higher vaccine dosage to target immunogenicity in these patients was frequently encountered [9].

Currently, there are several vaccines approved or undergoing phase III clinical trials for SARS-CoV-2 infection [10]. In general, live attenuated vaccines are suggested to be avoided due to the dysregulated immune system in dialysis patients. Both the mRNA vaccines BNT (Pfizer-BioNTech) and mRNA-1273 (Moderna) and the replication-defective viral-vectored vaccines, such as ChAdOx1 nCoV-19 (Oxford-AstraZeneca, AZ), are considered safe for use in dialysis patients [10]. The efficacy of vaccines is generally lacking in immunodeficient and dialysis patients. Recent data regarding the response rate toward COVID-19 vaccination in dialysis varied from 29.6% to 96.4% [11,12,13]. Grupper et al., used the immunoassay to quantify the antibodies against the receptor-binding domain (RBD) of the S1 subunit of the spike protein of SARS-CoV-2 (anti-RBD-Ab), demonstrating the suboptimal humoral response to the BNT vaccine in the dialysis patients [14]. A short report by Ducloux et al., suggested a need for a third dose of BNT to boost immune response in dialysis patients [15].

The second vaccine was delayed to 10 weeks after the first dose to achieve maximum population coverage in Taiwan. A pooled analysis of four randomized AZ trials showed that a single dose is efficacious, and the delay may improve efficacy [16]. It is vital to understand the immune response of CKD patients to guide the current and future vaccine dosing strategies in this vulnerable group. This study aims to establish the immune responses to the AZ vaccine in chronic dialysis patients. We followed the antibody responses against the RBD of the spike protein at the designated time points before and after vaccinations. We also studied for clinical parameters associated with delayed immune responses.

## 2. Materials and Methods

### 2.1. Study Design

Chronic dialysis is defined as patients receiving hemodialysis (HD) or peritoneal dialysis (PD) for more than three months. Three hundred and fifteen chronic dialysis patients aged greater than eighteen years were eligible to participate in the present study in the two Taipei Medical University-affiliated hospitals, Taipei Medical University-Wan Fang Hospital (WFH, a tertiary care hospital located in metropolitan Taipei city) and Taipei Medical University Hospital (TMUH, a tertiary care hospital located in the center of metropolitan Taipei city). According to the local regulation, all participants received the two recommended doses of AZ vaccines with a ten weeks interval between the first and second doses. There was only one patient under immunosuppressive therapy, a 51-year-old man with systemic lupus erythematosus under prednisolone 5 mg and mycophenolate mofetil 250 mg daily.

Following the institutional review board approval (TMU-eIIRB N202106049), we obtained informed consent from the participants to draw blood at the beginning of the dialysis session for the HD group and venous blood samples for the PD group. Immunogenicity assessment was determined using a chemiluminescent microparticle immunoassay (SARS-CoV-2 IgG II Quant assay on an ARCHITECHT analyzer, Abbott) to quantify IgG antibodies from the patient’s plasma. The laboratory protocol was followed by a method previously published by Walsh et al. [17]. A value ≥ 50 arbitrary units per milliliter (AU/mL) was considered to be a vaccine responder. Four blood draws were designed to evaluate the humoral response of patients after vaccination. T0, 0–7 days before the first dose of vaccine. T1, 14–20 days after the first dose of vaccine, T2, day 0–4 before the second dose of vaccine, and T3, day 21–35 after the second dose of vaccine. To ensure all patients met our quality control medical care, we blood tested every HD and PD patient every month. (Blood tests include complete blood count (CBC), blood urea nitrogen (BUN), creatinine (Cr), sodium (Na), potassium (K), calcium (Ca), phosphorus (Pi), intact parathyroid hormone (intact PTH), albumin, ferritin, triglyceride (TG), cholesterol, glutamic oxaloacetic transaminase (GOT), glutamic pyruvic transaminase (GPT), and total and direct bilirubin). Dialysis patients’ demographic details were obtained from their electronic medical records. A weekly rapid antigen screen was performed on every patient in both centers to ensure no asymptomatic infected patients in the dialysis units.

### 2.2. Flow Cytometry Analysis

After isolation of peripheral blood monocytes (PBMCs) from patients’ blood samples, PBMCs were submitted to flow cytometry immunofluorescence assay using the Attune Nxt Flow Cytometer (Thermo Fisher Scientific, Waltham, MA, USA). After washing with phosphate-buffered saline (PBS), Fc receptors were initially blocked using Human FcR blocking reagent (Miltenyi Biotec, Bergisch Gladbach, Germany) for 30 min at 4 °C, followed by cell surface labeling by specific primary antibodies. The following antibodies were adapted into multiple panels: CD19, CD3, CD4, Cd8, CD56, CXCR3, CD69, and IgG. (Thermo Fisher Scientific, Waltham, MA, USA). Results were illustrated as the percentage of positive cells from the antibody of interest to the isotype control antibody.

### 2.3. Statistical Analysis

All data were summarized and displayed as mean ± standard deviation (SD) for the continuous variables, the number of patients, and the percentage for each group for categorical variables. The chi-square statistic was used to assess the statistical significance between groups for all categorical variables. Continuous variables were first tested for normal distribution using the Kolmogorov–Smirnov test and quantile-quantile plots; then, parameters were compared by using a t-test if normally distributed or by the Kruskal–Wallis/Mann–Whitney U test if not normally distributed. A generalized additive model (GAM) (with spline) incorporated subject-specific random effects, expressed as the logarithm of the odds (logit), and the optimal cut-off value was defined as a log-odds value of zero [18].

We fitted binary logistic regression models for the study group’s lower quartile risk, adjusted for covariates. We used a line graph to depict antibody levels of cohorts to demonstrate differences in the designated timeline after vaccines. *p*-value < 0.05 was considered statistically significant for all analyses. IBM SPSS Statistics for Windows, version 25 (IBM Corp., Armonk, NY, USA) was used for all statistical analyses.

## 3. Results

The clinical characteristics and laboratory data of patients for the present study are summarized in Table 1. Three hundred and eight patients had completed two vaccine doses for the final evaluation. None of our patients had positive SARS-CoV-2 rapid antigen tests during the three-month study period. There were 269 HD and 39 PD patients who participated in the present study. Complete demographic, clinical, and laboratory data were presented in the Supplementary Table S1. In summary, HD patients were significantly older (66.2 ± 12.37 vs. 60.9 ± 11.61), had higher serum albumin, potassium, and sodium levels than PD patients (3.88 ± 0.38 vs. 3.57 ± 0.31, 4.6 ± 0.73 vs. 4.0 ± 0.67, 137.0 ± 8.7 vs. 133.9 ± 4.1, respectively).

### 3.1. Anti-RBD Antibody Levels at Different Time Points

**Figure 1:**
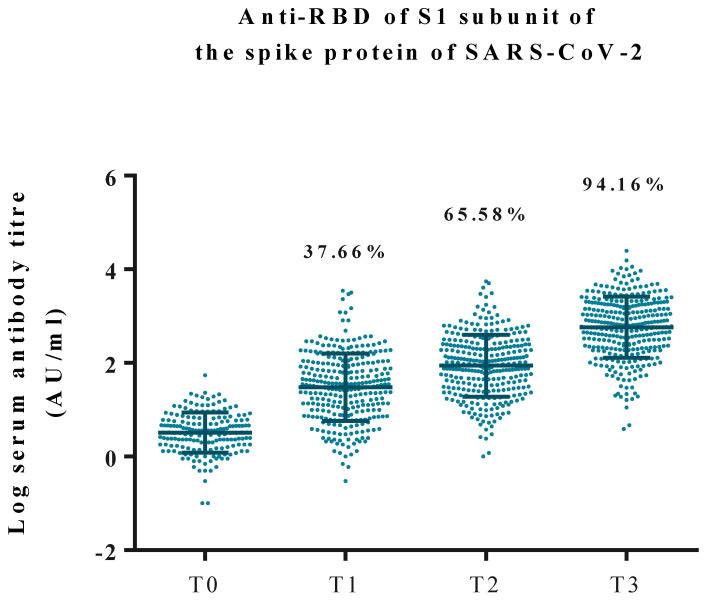
Log10 value of antibody titers against the RBD of the S1 subunit of the SARS-CoV-2 spike protein in arbitrary units (AU)/mL. Data on the graph represents the percentage of participants’ antibody titers greater than 50 AU/mL. Abbreviation: RBD, receptor-binding domain.

Figure 1 shows the antibody levels of all 308 patients in the four test time points (data represents on log10 value of serum anti-RBD titers). At T0, all patients had antibodies levels <50 AU/mL (average antibody 4.5 ± 5.8 AU/mL, log10 value = 0.51 ± 0.43 AU/mL). At T1, only 37.66% of participants (116 of 308 participants) had positive antibodies (average antibody 120.6 ± 371.2 AU/mL, log10 value = 1.48 ± 0.72 AU/mL). At T2, ten weeks after the first vaccine, 65.58% of participants became seropositive (204 of 308 participants, average antibody 266.1 ± 605.5 AU/mL, log10 value = 1.94 ± 0.66 AU/mL). At T3, four weeks after the second dose of the vaccine, 18 participants (5.84%) remained unresponsive to vaccines (sero-naïve, SN, average antibody 19.23 ± 11.58 AU/mL). The average antibody of all patients was 1347.4 ± 2459.0 AU/mL (log10 value = 2.76 ± 0.65 AU/mL). The average anti-RBD-Ab levels at T3 in HD patients were 1342.0 ± 1894.0 AU/mL and the PD patients were 2236.0 ± 4592.0 AU/mL, HD vs. PD, *p* = 0.394 had no statistical significance. (Supplementary Table S1).

### 3.2. Clinical Factors Associated with Poor Immunogenicity of Dialysis Patients

#### 3.2.1. Age Was the Essential Factor Associated with the Vaccine Response

Age was the essential contributory factor associated with the AZ vaccine response (OR 0.948, *p* = 0.015; OR 0.921, *p* = 0.003 univariable and multivariable analysis, respectively). Using multivariable analysis identified both hemoglobin (Hb) and diabetes mellitus (DM) as were other two significant factors associated with sero-responsiveness (Table 2).

#### 3.2.2. Multinominal Logistic Regression to Evaluate Clinical Factors Associated with Vaccine Response

Another statistical model was established by dividing antibody levels at T2 into quartiles using multinominal logistic regression to evaluate the potential clinical factors associated with the immune response to the AZ vaccine (Table 3). Age appeared to be the only significant factor associated with different quartiles of antibody levels. Bodyweight and Hb were statistically significant factors between the first and fourth quartile antibody levels. There was no statistical significance despite the average antibody levels being higher in PD than HD patients.

### 3.3. Anti-RBD Antibody Levels at the Different Age Groups

**Figure 2:**
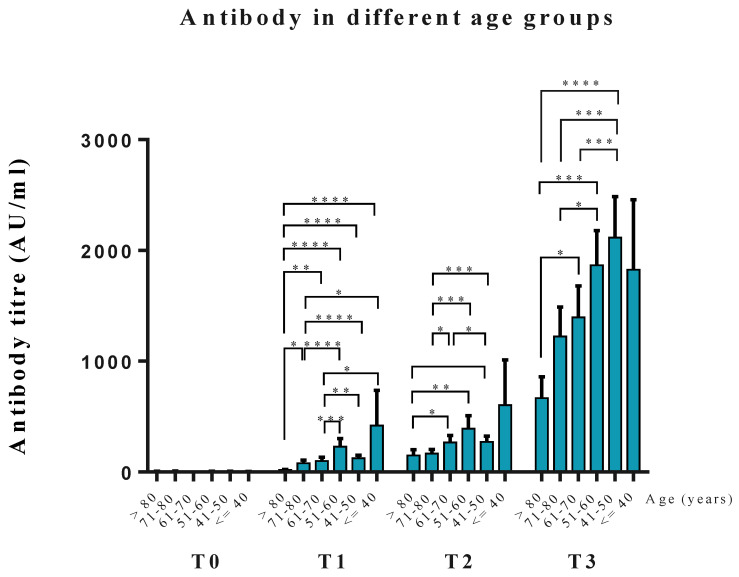
Anti-RBD antibody levels at the four-time points in the six age groups. * *p* < 0.05, ** *p* < 0.01, *** *p* < 0.005, **** *p* < 0.001.

**Figure 3:**
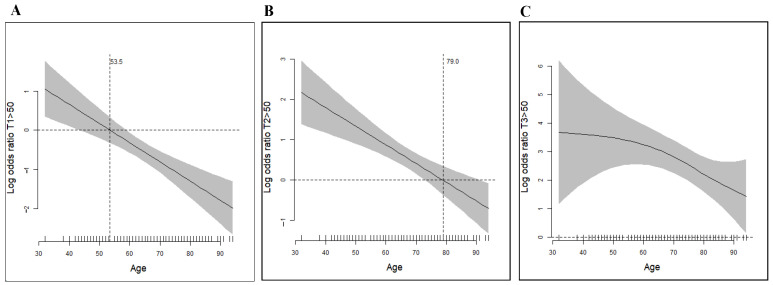
GAM plots show the negative association of the anti-RBD antibody responses with the age of the participants. (**A**) At T1, two weeks after the first dose of vaccine, participants with age less than 53.5 years had a positive odd ratio in developing positive anti-RBD antibody response. (**B**) At T2, ten weeks after the first dose of vaccine, participants with age less than 79.0 years had a positive odd ratio in developing positive anti-RBD antibody response. (**C**) At T3, four weeks after the second dose of vaccine, most participants across all age groups developed positive anti-RBD antibody response. Abbreviations: GAM, generalized additive models; RBD, receptor-binding domain.

We categorized patients into six groups according to age. Significant differences in antibody levels were found in different age groups in T1, T2, and T3 (Figure 2). At T3, 60 years seemed to be the threshold age to have different degrees of antibody responses. A GAM (generalized additive model) plot was used to find the adequate cut-point value to age to predict the probability of antibody response to the vaccine. At T1, 53.5 years of age negatively predicted antibody response after receiving the AZ vaccine (Figure 3A). At T2, 79.0 years of age showed a cut-point value to predict positive antibody response at ten weeks after the first dose of vaccine (Figure 3B). Most patients developed positive immune responses using the GAM plot at T3 (Figure 3C).

### 3.4. Cellular Dynamics Underlying B Cell Response to the Vaccine

**Figure 4:**
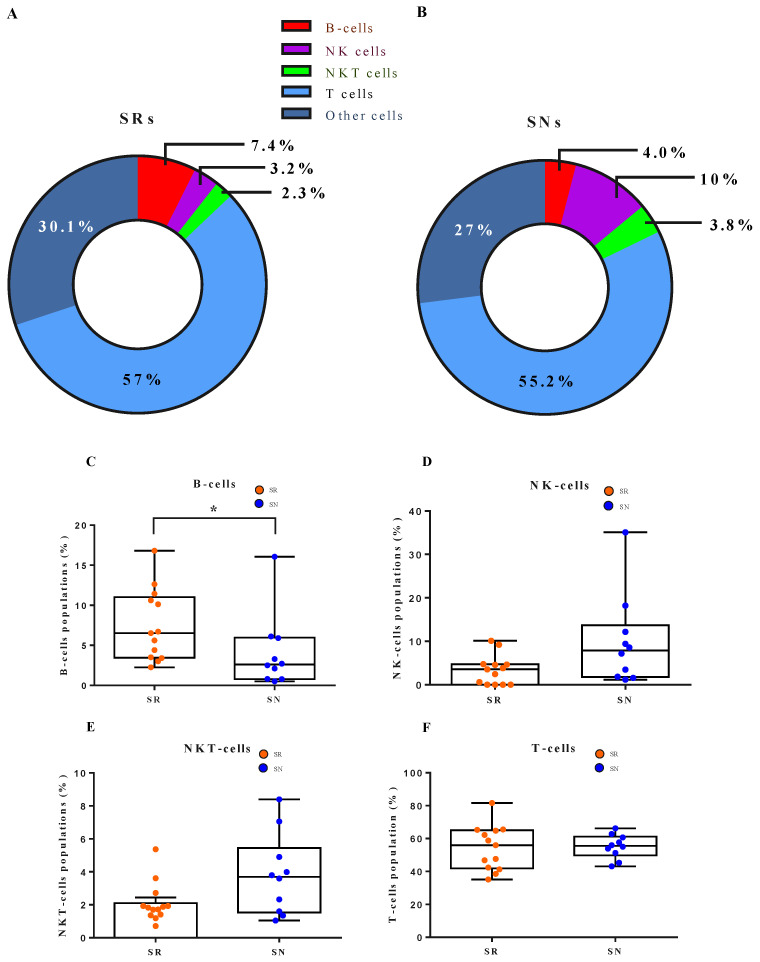

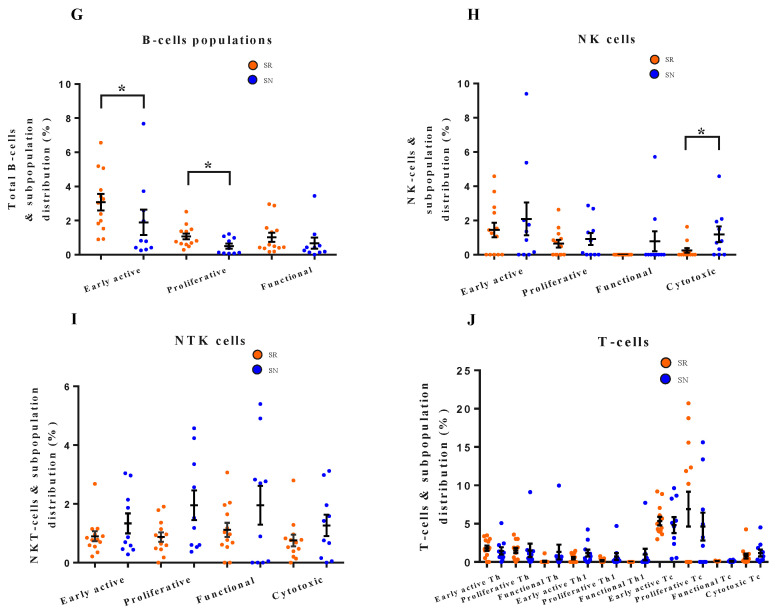
The humoral and cell-mediated immune responses of SNs compared the age- and sex-matched SRs after two doses of vaccines. The flow cytometry analysis elicits both humoral and cell-mediated responses by enumeration of lymphocyte cellular markers and proliferation assays. (**A**–**F**) After two doses of vaccines, responders showed significantly higher B-cells than sero-naïve participants in their PBMCs. In contrast, SNs had higher NK cells and NKT cells than SRs, but no statistical significance was reached. (**G**–**J**) SRs showed significantly higher numbers in early active and proliferative B-cells than SNs. In contrast, SNs had significantly higher cytotoxic NK cells. Abbreviations: PBMCs, peripheral blood monocytes; NK, natural killer cells; NKT, natural killer T cells; SNs sero-naïve participants; SRs, sero-responders. * *p* < 0.05.

Ten of eighteen SN patients agreed to receive flow cytometry analysis. Thirteen of the age- and sex-matched sero-responders (SRs) agreed to receive cytometry analysis and the results compared with the SN patients. Figure 4A,B illustrated the percentage of positive cells from the antibody of interest to the isotype control antibody. The SRs showed a higher B-cell population than the SNs (SR 7.4% vs. SN 4%). In particular, early active and proliferative B-cells were found to have a significantly higher population in the SNs’ PBMC (Figure 4G). Conversely, SNs had significantly higher cytotoxic natural killer (cNK) cells (Figure 4H).

## 4. Discussion

This study describes the IgG antibody response to the RBD of spike protein S1 subunit following the AZ vaccines in chronic dialysis patients. We found that 94.16% of chronic dialysis patients developed a substantial humoral response following the second dose of vaccine despite a delayed response after the first vaccine. Only 18 chronic dialysis patients had IgG levels below 50 AU/mL following the second dose of vaccine (sero-naïve, SNs). Among these 18 patients, only one was under immunosuppressive therapy. A 51-year-old man with systemic lupus erythematosus received prednisolone 5 mg and mycophenolate mofetil 250 mg daily. All other SNs had not received any immunosuppressive therapies. Patients’ age appeared to be a significant factor associated with an immune response to the vaccine. An inverse relationship was found between age and IgG levels. At T1, patients’ age younger than 53.5 years had a better response to the AZ vaccine than their counterparts. At T2, patients less than 79 years of age would have better sero-response (Figure 3A,B). At T3, a statistical difference was found in the mean Age of SNs vs. SRs (71.8 ± 13.81 vs. 65.1 ± 12.20 years, respectively). A similar finding of age-related lower anti-RBD response to a BNT162b2-mRNA vaccine was reported by Bachelet et al., recently [19].

Grupper et al., reported that the average anti-SARS CoV2 S1 unit of spike protein antibody was 3946 AU/mL in maintenance dialysis patients at a median of 30 days after the second dose of the BNT vaccine [20]. Using the same serological testing assay (Abbott), the average antibody levels in our patient at 28 days after the second AZ vaccine was 1347.4 ± 2459.0 AU/mL. An earlier study by Folegatti et al., using a multiplexed immunoassay (Meso Scale Discovery Multiplexed Immunoassay [MIA] RBD) reported that the median IgG level of their 482 healthy participants was 3182.5 AU/mL (IQR, 1426.3, 6800.4) at 28 days after the first AZ vaccine (our patients, median 30.30, 93.65 IQR 7.45, 98.1; 29.10, 248.5 AU/mL at T1 and T2, respectively). Whether different immunoassay measurements for anti-RBD-Ab would be interchangeable remains further confirmation. The same study tested nine participants on day 28 after the second vaccine, and the median IgG antibody was 16,825.4 (IQR 13,118.9, 20,937.9) [21]. In the same period after the second dose of vaccine, our patients’ median IgG antibody was 657.1 (IQR 212.5, 1788).

Hemoglobin was also the significant factor associated with sero-response by multivariable and multinominal logistic regression. Hassan et al., demonstrated that higher Hb levels were associated with a better Engerix-B (HBV) vaccine response in pre-dialysis CKD patients [22]. Moreover, published data showed in the dialysis patients that elderly age and lower Hb were risk factors in developing a lower response to the influenza vaccine [23,24]. Inflammation and nutritional status have been known to relate to the immune response to the vaccine [25]. Anemia is commonly associated with inflammation and the nutritional status of dialysis patients [26]. The present study did not have enough clinical information to assess the influence of inflammatory or nutritional parameters on seroconversion.

DM was also the critical factor associated with poor antibody response in our study. Currently, further evidence remains to tackle whether diabetic patients have a suboptimal response to the SARS-CoV2 vaccine. However, following the HBV vaccine’s experience, Alavian et al., demonstrated that the HBV seroprotection rate in diabetic CKD patients was significantly lower than in non-diabetic CKD patients [27].

Different dialysis modalities may have different seroconversion responses. Khan et al., showed that PD patients had a lower seroconversion rate after HBV vaccination than HD patients [28]. However, a meta-analysis showed a different result by pooling 1211 dialysis patients from 14 clinical trials showed there is no significant link between dialysis mode and sero-response to HBV vaccine in the dialysis population [29]. In the present study, the antibody levels were higher in PD than in HD patients (2236.0 ± 4592.0 vs. 1342.0 ± 1894.0) (Supplementary Table S1). However, no statistical significance was found between the groups. A higher number of HD than PD participants probably accounted for the lack of statistical significance in the present study. However, whether dialysis modality accounts for immune response to the SARS-CoV2 vaccine may need a more extensive scale study.

Vaccine triggering immune response involves a complex cellular dynamic to activated B-cell response. Antigen and B-cell receptors interaction initiate the early B-cell proliferation. Following the proliferative phase, early B-cells differentiated into the short-lived plasma cells (SLPC), germinal center (GC) cells, and memory B-cells. GCs give more SLPCs, memory Bs, and long-lived plasma cells (LLPC) in response to the subsequent antigen stimulation [30]. The flow cytometry disclosed that the SNs had a significantly depressed level of B-cells. In particular, both early active and proliferative B-cells were significantly lower in the SNs (Figure 4). Our finding is compatible with the report from the mRNA vaccine BNT162b2 study performed by Rincon-Arevalo et al. [31].

Our patients were primarily tolerable to the AZ vaccine. Most patients had minor symptoms and recovered entirely within a week (Figure S1). Only two patients experienced shortness of breath immediately after the vaccine. However, no clinical significance was found.

Our study was limited by two local centers’ experience involving an average of a relatively older age population. There were no age and gender-matched healthy individuals from the local data to compare our patients’ humoral responses. However, we had the advantage of following the serological response to the AZ vaccine in non-infected dialysis patients. The weekly rapid antigen screens helped us identify the concealed COVID-19 infection patients. Any potentially infected patients would be excluded from our analysis.

## 5. Conclusions

Age appeared to be the essential factor associated with humoral response in chronic dialysis patients after the ChAdOx1 nCoV-19 (Oxford-AstraZeneca) vaccination. No CKD-specific factors hinder the humoral response (as demonstrated in the present study). Although the present study did not include the healthy control subjects, previous reports suggest a similar percentage of humoral response in healthy individuals, such as in the CKD patients in the current study [32]. A depressed B-cells response was found in a subset of patients with a suboptimal anti-RBD-Ab response. The implication of this finding will require further immunological investigations.

## Figures and Tables

**Table 1:** Demographic, clinical, and laboratory data of chronic dialysis patients.

	All Patients (*n* = 308)
Age ± SD (years)	65.5 ± 12.38
HD/PD patient numbers	269/39
Sex (Male/Female)	189/119
Body weight (kg)	63.9 ± 13.49
BMI (kg/m^2^)	24.16 ± 4.11
Kt/V (HD/PD)	1.53 ± 0.23/2.06 ± 0.24
URR/WCC (HD/PD)	72.90 ± 5.32/61.32 ± 16.39
Albumin (g/dL)	3.84 ± 0.38
Dialysis vintage (Months)	74.13 ± 69.04
Ferritin (ng/mL)	437.85 ± 348.88
WBC (×10^3^/μL)	4.31 ± 3.46
Hemoglobin (g/dL)	10.34 ± 1.21
Platelet (10^3^/μL)	178.86 ± 68.11
Sodium (mmol/L)	136.65 ± 8.33
Potassium (mmol/L)	4.54 ± 0.75
Calcium (mg/dL)	9.1 ± 0.81
Phosphate (mg/dL)	5.02 ± 1.45
Intact PTH (pg/mL)	409.52 ± 400.71
Triglyceride (mg/dL)	175.68 ± 131.72
Cholesterol (mg/dL)	151.32 ± 41.01
GOT (U/L)	15.78 ± 8.97
GPT (U/L)	14.29 ± 9.68
Total Bilirubin (mg/dL)	0.45 ± 0.19
Direct bilirubin (mg/dL)	0.11 ± 0.10
DM (%)	152 (49.35%)
Hypertension (%)	247 (80.19%)

Abbreviations: BMI, body mass index; DM, diabetes mellitus; GOT, glutamic oxaloacetic transaminase; GPT, glutamic pyruvic transaminase; HD, hemodialysis; intact PTH, intact parathyroid hormone; Kt/V, quantifying hemodialysis and peritoneal dialysis treatment adequacy, K, dialyzer clearance of urea; t, dialysis time; V, the volume of distribution of urea. PD, peritoneal dialysis; URR, urea reduction ratio; WBC, white cell counts; WCC, weekly creatinine clearance.

**Table 2:** Clinical factors associated with poor immunogenicity of dialysis patients.

Variables	Non-Responders vs. Responders			
	Univariable		Multivariable	
	OR (95% CI)	*p* Value	OR (95% CI)	*p* Value
Age (Years)	0.948 (0.907–0.990)	0.015 *	0.921(0.874, 0.972)	0.003 *
Sex	2.124 (0.676–6.673)	0.197		
BMI (kg/m^2^)	0.982 (0.880–1.096)	0.744		
BW (kg)	0.990 (0.958–1.024)	0.567		
Dialysis modality (HD/PD)	2.127 (0.336–13.448)	0.423		
GPT (U/L)	0.974 (0.939–1.010)	0.153		
K (mmol/L)	1.075 (0.556–2.081)	0.830		
TG (mg/dL)	1.002 (0.997–1.007)	0.351		
Hb	1.453 (0.979, 2.158)	0.064	1.756 (1.137, 2.713)	0.011 *
WBC (×10^3^/μL)	0.982 (0.958–1.007)	0.159		
DM	0.542 (0.195–1.504)	0.239	0.291 (0.089, 0.949)	0.041 *
Kt/V	4.322 (0.647, 28.897)	0.131		
URR	1.006 (0.952, 1.064)	0.822		
Dialysis vintage (months)	1.001 (0.994–1.008)	0.793		

Abbreviations: BMI, body mass index; BW, body weight; HD, hemodialysis; PD, peritoneal dialysis; K, potassium; Kt/V, quantifying hemodialysis and peritoneal dialysis treatment adequacy, K, dialyzer clearance of urea; t, dialysis time; V, the volume of distribution of urea. TG, triglyceride; Hb, hemoglobin; WBC, white cell count; DM, diabetes mellitus; URR, urea reduction ratio. * *p* < 0.05.

**Table 3:** Model of antibody levels divided into quartile against clinical variables (Ref. 4th quartile).

	1st Quartile (<212.5 AU/mL)			2nd Quartile (212.5–657.0 AU/mL)			3rd Quartile (657.1–1788 AU/mL)		
	OR	95% CI	*p*-Value	OR	95% CI	*p*-Value	OR	95% CI	*p*-Value
Age (yrs)	1.11	1.06–1.15	0.0001 ***	1.06	1.02–1.10	0.001 **	1.04	1.01–1.08	0.013 **
Sex	0.86	0.30–2.49	0.784	1.16	0.43–3.16	0.766	0.60	0.22–1.68	0.335
BW (kg)	1.08	1.00–1.17	0.038 *	1.01	0.95–1.09	0.71	1.06	0.99–1.14	0.111
Alb (g/dL)	1.17	0.38–3.61	0.791	1.12	0.37–3.33	0.843	0.99	0.34–2.95	0.990
K (mmol/L)	1.24	0.74–2.05	0.414	1.10	0.68–1.79	0.689	0.96	0.59–1.58	0.877
TG (mg/dL)	0.99	0.99–1.01	0.129	0.99	0.99–1.01	0.206	1.01	0.99–1.01	0.735
GPT (U/L)	0.98	0.95–1.02	0.343	0.97	0.93–1.01	0.076	0.97	0.93–1.01	0.108
WBC (×10^3^/μL)	1.08	0.97–1.21	0.155	1.05	0.93–1.18	0.458	1.08	0.96–1.20	0.189
Hb (g/dL)	0.62	0.45–0.87	0.005 *	0.81	0.60–1.10	0.176	0.82	0.60–1.12	0.217
DM	0.88	0.42–1.87	0.743	1.75	0.80–3.47	0.170	0.88	0.43–1.83	0.741
Dialysis vintage (months)	0.99	0.99–1.01	0.198	1.01	0.99–1.01	0.420	0.99	0.99–1.01	0.229
Kt/V	1.48	0.36–6.08	0.589	1.14	0.30–4.38	0.846	0.37	0.09–1.61	0.185

Abbreviations: Alb, albumin; BW, body weight; GPT, glutamic pyruvic transaminase; K, potassium; Kt/V, quantifying hemodialysis and peritoneal dialysis treatment adequacy, K, dialyzer clearance of urea; t, dialysis time; V, the volume of distribution of urea. TG, triglyceride; WBC, white cell count; Hb, hemoglobin; DM, diabetes mellitus. * *p* < 0.05, ** *p* < 0.01, *** *p* < 0.005.

## Supplementary Materials

The following supporting information can be downloaded at: https://www.mdpi.com/article/10.3390/vaccines10020338/s1, Figure S1: Symptoms of the participants after the first dose of ChAdOx1 nCoV-19 vaccine; Table S1: Clinical characteristics of chronic dialysis patients, HD vs. PD.
