# Supplementary material for: The Humoral Immune Response of the ChAdOx1 nCoV-19 Vaccine in Maintenance Dialysis Patients without Prior COVID-19 Infection
Source: Vaccines (Basel). 2022 Feb 21;10(2):338. doi: 10.3390/vaccines10020338 (PMC8879203; doi:10.3390/vaccines10020338)
Supplement: Supplementary file 1 [file vaccines-10-00338-s001.zip › vaccines-1548232-supplementary.pdf]

Supplementary data

Table S1 Clinical characteristics of chronic dialysis patients, HD vs. PD

	HD(N=269)	PD(N=39)	P value
Age±SD (years)	66.2±12.37	60.9±11.61	0.019*
Sex (Male/Female)	168/101	21/18	0.304
Body weight (kg)	63.7±13.71	65.6±11.88	0.301
BMI (kg/m^2)	24.0±4.14	25.1±3.78	0.067
Kt/V (HD/PD)	1.60±0.29	2.07±0.24	
URR/WCC (HD/PD)	72.9±5.32	61.1±16.39	
Albumin (g/dL)	3.88±0.38	3.57±0.31	0.0001***
Dialysis vintage (Months)	76.5±70.89	56.2±52.19	0.097
Ferritin (ng/mL)	437.8±348.9	412.7±523.0	0.106
WBC (x10^3/µl)	6.29±1.94	7.16±1.99	0.014*
Hemoglobin (g/dL)	10.3±1.06	10.3±1.97	0.414
Platelet (10^3/µL)	172.2±59.1	221.9±101.7	0.0002***
Sodium (mmol/l)	137.0±8.7	133.9±4.1	<0.0001****
Potassium (mmol/l)	4.6±0.73	4.0±0.67	<0.0001****
Calcium (mg/dl)	9.1±0.8	9.2±0.8	0.219
Phosphate (mg/dl)	5.0±1.4	5.1±1.7	0.730
Intact PTH (pg/ml)	401.5±404.3	464.4±375.3	0.064
Triglyceride (mg/dl)	176.3±133.6	171.2±119.2	0.834
Cholesterol (mg/dl)	149.9±39.6	161.1±49.4	0.288
GOT (U/l)	15.5±9.1	17.7±8.2	0.041*
GPT (U/l)	13.5±9.3	20.1±10.5	<0.0001****
Total Bilirubin (mg/dl)	0.45±0.19	0.47±0.16	0.469
Direct bilirubin (mg/dl)	0.11±0.10	0.11±0.05	0.550
DM	136	16	0.268
Hypertension	210	37	0.026*
Anti-RBD Ab (AU/ml): T3	1342±1894.0	2236.0±4592.0	0.394
T2	274.7±637.2	209.2±323.5	0.589
T1	132.1±395.1	39.78±54.28	0.086
T0	3.9±4.3	7.0±9.6	0.015*

Abbreviations: BMI, body mass index; DM, diabetes mellitus; GOT, glutamic oxaloacetic transaminase; GPT, glutamic pyruvic transaminase; HD, hemodialysis; intact PTH, intact parathyroid hormone; Kt/V, quantifying hemodialysis and peritoneal dialysis treatment adequacy, K, dialyzer clearance of urea; t, dialysis time; V, the volume of distribution of urea. PD, peritoneal dialysis; URR, urea reduction ratio; WBC, white cell counts; WCC, weekly creatinine clearance.

Figure S1

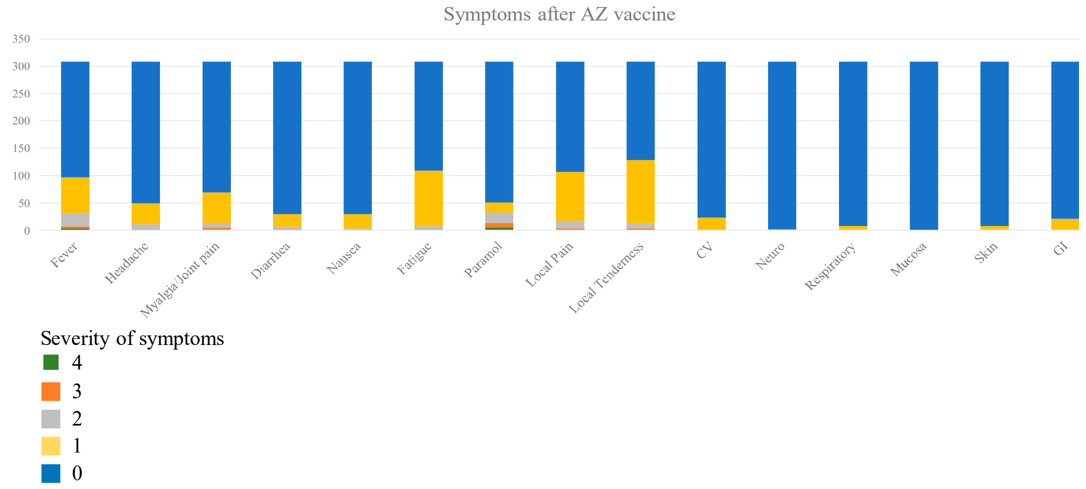

Figure S1. Symptoms of the participants after the first dose of ChAdOx1 nCoV-19 vaccine. Different color represents different severity of symptoms.
